# Unmet Information Needs of Spanish Female Breast Cancer Survivors on Chemical Pollutants: A Cross-Sectional Mixed-Method Study

**DOI:** 10.3390/toxics14060456

**Published:** 2026-05-23

**Authors:** Laura García-Molina, Bibiana Navarro-Matillas, Blanca Riquelme-Gallego, Marina Zenobia Molina-Fernández, José Expósito, Juan Pedro Arrebola, Piedad Martin-Olmedo

**Affiliations:** 1 Department of Preventive Medicine and Public Health, Faculty of Medicine, University of Granada, Avenida Doctor Jesús Candel Fábregas, 11, 18016 Granada, Spain; lgarmol@ugr.es (L.G.-M.); blanca.riquel@gmail.com (B.R.-G.); jparrebola@ugr.es (J.P.A.); 2 Department of Public Health, Environmental Health, and Citizen Services, Escuela Andaluza de Salud Pública, Cta. del Observatorio, 4, Beiro, 18011 Granada, Spain; bibiana.navarro.matillas@gmail.com; 3 Radiation Oncology Department, Virgen de las Nieves University Hospital, Avenida de las Fuerzas Armadas 2, 18014 Granada, Spain; marinazenobia.ort@gmail.com (M.Z.M.-F.);; 4 Instituto de Investigación Biosanitaria (ibs.GRANADA), 18012 Granada, Spain

**Keywords:** breast cancer, environmental exposure, chemical pollutants, behavior, information needs

## Abstract

Breast cancer (BC) remains the most prevalent malignancy among women. Prolonged exposure to chemical pollutants has been identified as one of its potential risk factors. Despite increased access to health information, important informational gaps persist regarding chemical exposure. This concern is particularly pronounced among female BC survivors due to their greater vulnerability. This study investigated unmet information needs related to chemical exposure among female BC survivors from Granada (Spain). The data were collected through 150 semi-structured interviews and focus groups between March 2023 and April 2024 and analyzed using the Framework Method. Most participants demonstrated limited awareness regarding BC and its potential association with chemical exposure. Nevertheless, 78.67% of them expressed concerns regarding the issue after diagnosis and anxiety surrounding the lack of reliable information from trusted sources on recurrence prevention. These findings highlight the need for public health communication that informs and empowers protective behavior.

## 1. Introduction

Breast cancer (BC) is the most common cancer in women globally, with a higher incidence than in men [1,2]. According to the Spanish Society of Medical Oncology (SEOM), breast cancer accounts for 28.9% of all cancers diagnosed in women and continues to rise, with 36,395 new cases reported in Spain in 2024—over 2000 more than in 2022. In the province of Granada alone, 5462 new cancer cases were reported among the adult population in 2024, with breast, colorectal, uterine corpus, and cutaneous melanoma being the most frequently diagnosed types [3]. In parallel, BC survival rates have improved due to advances in medical science, early detection, and combined treatments like chemotherapy, hormone therapy, and radiotherapy, leading to a growing community of BC survivors with distinct care needs [4]. In Spain, the survival rates following a BC diagnosis are currently estimated at over 88% at five years, surpassing the European average of 83% [3].

Among the risk factors associated with BC development, two major categories of determinants can be identified. The first group comprises potentially modifiable factors, including lifestyle-related risks such as breastfeeding history, tobacco use, excessive alcohol consumption, obesity, and environmental exposure to chemicals—over which individuals have limited control unless provided with accurate and accessible information [5,6]. The second group encompasses non-modifiable factors, such as age, family history of cancer, late onset of menopause [7,8].

Many of these chemicals are usually generated through anthropogenic processes, e.g., as by-products of industrial activity or agricultural practices. The list includes ambient air pollutants, pesticides, pharmaceutical compounds, and pollutants that may enter the food chain via air deposition or water and soil contamination [9], as well as substances found in hygiene and personal care products, cosmetics, textiles, and dyes [10,11]. Among the most extensively documented chemical threats to human health are persistent organic pollutants (POPs), such as dioxins, polychlorinated biphenyls (PCBs), phthalates, bisphenol A (BPA), parabens, triclosan, perfluorooctanoic acid (PFOA), perfluorooctane sulfonate (PFOS) and per- and polyfluoroalkyl substances (PFASs) [10,11,12]. These chemicals are ubiquitous in the environment and bioaccumulative, so once they overcome the absorption barriers of the human body via oral, dermal or inhalation exposure routes, they can generate prolonged impact on different human organs, in many cases with estrogenic activity [13,14]. High-quality scientific evidence supports that repeated exposure to endocrine-disrupting chemicals (EDCs) increases the risk of problems in reproductive health, diabetes, obesity, metabolic disorders, thyroid homeostasis [15,16] and several cancers including BC [11,14,17,18]. A study conducted in 2023 examined the association between cumulative environmental threats—particularly in urban settings—and the increased incidence of BC. The findings underscore the impact of urban exposure to multiple environmental chemicals on public health, highlighting the need for urban planning and environmental policies to account for these risks in order to reduce the associated burden of disease [7].

Multiple studies have acknowledged the need to develop lifestyle interventions for BC survivors as a critical component in minimizing recurrences and comorbidities [19,20]. Beyond clinical outcomes, BC exerts substantial physical, psychological, and social burdens on the affected women, contributing to a strong demand for counseling and relevant information about the specific characteristics of their cancer and treatment, body image and sexuality, or options for enhancing their quality of life [4,21]. With the increasing availability of information and communication technologies, survivors now have more opportunities than ever to actively participate in their health and care, supported by resources tailored to their evolving needs.

In this context, “unmet and information needs” have been described as the gaps cancer survivors perceive in the level of service and information required to achieve optimal well-being after diagnosis and treatment [22]. Understanding these needs is key for improving patient-centered care, which will enhance outcomes, their quality of life, and reduce pressure on social and healthcare services [4,21,22].

Although numerous studies and systematic reviews have explored the unmet informational needs of cancer survivors—particularly in areas such as lifestyle modifications [4,20,22]—there is still a notable lack of evidence addressing breast cancer survivors’ need for information about exposure to chemicals and their potential link to the disease.

This study aims to explore perception and unmet information needs regarding exposure to environmental risk factors—especially to chemicals—and their associated health risks among female BC survivors of Granada (Spain). The study focuses on potentially inadvertent exposures to chemicals present in different environmental media (e.g., air and water) as well as in everyday consumer and personal care products, where the presence of potentially harmful substances is often not transparent to users. Changes in lifestyle and behavior after the patient’s diagnosis are also analyzed, identifying this population’s information needs to support protective measures.

## 2. Materials and Methods

### 2.1. Design and Study Population

A cross-sectional mixed-method study was conducted between March 2023 and April 2024 to analyze risk perception and unmet information needs regarding environmental exposure to chemicals. This study included an equitable and non-discriminatory sample of 150 female BC survivors receiving follow-up care at the oncology service of a University Hospital in Granada (Spain). All participants had completed active breast cancer treatment and were clinically stable at the time of study inclusion. Follow-up care consisted of routine post-treatment visits aimed at recurrence surveillance and/or the management of treatment-related side effects, in accordance with standard survivorship care practices within the Spanish National Healthcare System. The inclusion criteria for the target population were women aged 18 years or older undergoing BC follow-up care for at least one-year post-treatment, registered with the National Health System in Granada. The exclusion criteria were: (1) having active BC under treatment; (2) inability to understand and sign the informed consent form or to communicate with the study investigators.

The participant selection was carried out by trained personnel of the Oncology Service between March and July 2023. Participants were recruited using purposive, non-probabilistic sampling, consistent with the exploratory and qualitative nature of the study. Recruitment focused on information-rich cases, with sampling decisions guided by analytical adequacy and the stability of emerging themes rather than by participation rates or statistical representativeness. Accordingly, systematic information on individuals who declined participation was not collected, as comparisons between participants and non-participants were not considered analytically relevant to the interpretive aims of this article [23]. The city of Granada (population: 243,059) is surrounded by several commuter towns that together form the so-called Granada metropolitan area. Although these municipalities are officially designated as rural zones under the Spanish Law 45/2007 on the Sustainable Development of the Rural Environment (BOE-A-2007-21493), their populations are predominantly influenced by urban physical and environmental conditions. Many of the women included in this study resided in these commuter municipalities.

### 2.2. Data Collection

Between September and December 2023, 150 semi-structured interviews were conducted. The questionnaire was based on a previously applied instrument adapted for a population from the same area [24] and informed by internationally recognized topics used to assess unmet needs among cancer survivors and exposure to chemicals. The Quantitative questions mainly addressed common sociodemographic characteristics and individual lifestyle behaviors at the moment of the interview, including aspects related to diet, physical activity, sleeping habits, smoking, alcohol and drug habits, obstetric information, and auto-perceived health status. The open-ended questions in the qualitative section focused on the following topics: (1) awareness of environmental risk factors—particularly chemical pollutants—and their potential impact on human health; (2) information received on this topic during the course of their illness; (3) interest in being informed or guided on what type of products might contain chemicals and how to use them safely; (4) lifestyle changes made since the diagnosis; (5) interest in and ability to read labels for chemical information, such as cleaning products and household items (original questionnaire in Spanish and translated into English is available in Supplementary Materials S1, Section S1). The interviews lasted around 58 min. The interviews were tape-recorded and translated as faithfully as possible, preserving the original expressions and linguistic nuances.

At the end of each interview, participants were asked if they would be interested in taking part in a focus group meeting (FGM). The aim was to explore and collect additional insights into participants’ knowledge, perceptions, and practices related to the central topic of this study. Of the 150 participants, 62 agreed to be contacted. An email invitation was sent to 24 of these participants; purposively selecting people who indicated that they had changed some habits after diagnosis. Two FGMs, including 8 women per group, were conducted between January and April 2024 at the University of Granada in a relaxed setting to encourage open dialog and the expression of diverse viewpoints. The FGMs were moderated by a member of the research team and followed by two others who acted as observers (guidance questions available in Supplementary Materials S1, Section S2). All three specialists in qualitative methods introduced themselves and reiterated the study’s objectives and that information would be treated anonymously to foster a climate of trust. All sessions were tape-recorded and subsequently transcribed for discourse analysis. The duration of the FGMs did not exceed 120 min. Analysis of the two FGMs indicated thematic saturation, making additional sessions unnecessary.

### 2.3. Data Analysis

Data from the semi-structured questionnaires were analyzed using measures of central tendency and dispersion (median and 25th–75th percentiles) for quantitative variables, and absolute frequencies and percentages for categorical variables coded as yes/no or into multiple categories. Transcriptions of data from the open questions in the interviews and FGMs were managed by a trained member of the research team. Qualitative data from the open-ended interview questions and focus groups were analyzed using the Framework Method, with systematic coding in ATLAS.ti vs23 (https://atlasti.com) followed by thematic synthesis to generate themes and subthemes [25]. During the coding process, thematic synthesis was performed by grouping analytical categories that addressed similar topics, and percentages were calculated.

### 2.4. Ethical Considerations

The notification and handling of participants’ personal data complied with Spanish Organic Law 3/2018 on the Protection of Personal Data and Guarantee of Digital Rights, as well as with Regulation (EU) 2016/679 of the European Parliament and of the Council of 27 April 2016 (General Data Protection Regulation). The study protocol was approved by the Biomedical Research Ethics Committee of the Province of Granada (CEI/CEIM GRANADA) in January 2023. All participants were fully informed about the study objectives and procedures and provided written informed consent.

## 3. Results

### 3.1. Characteristics of the Study Population

The general demographic and lifestyle characteristics of the study population at the moment of the interview are provided in Table 1, while Table 2 and Table 3 show behavioral changes reported by participants following BC diagnosis.

The BC survivors in this study had a mean age of 59 years, a BMI of 25 kg/m^2^, and were three years post-diagnosis. Most participants were women with secondary or higher education (72.7%), living in rural areas (62.7%)—primarily within the Metropolitan region. While 80% reported following a balanced diet, 59.3% exhibited sedentary behavior. Regarding smoking habits, over half of the participants reported never having smoked, 11.33% were current smokers, and five individuals disclosed occasional marijuana use. Alcohol consumption divided the sample into non-consumers (50.67%) and occasional drinkers (40.67%). Dietary habits at the time of the interview reflected a moderate adherence to the Mediterranean diet, characterized by daily intake of dairy products, fruits, vegetables, and leafy greens; moderate consumption of eggs, meat, and legumes; and low intake of sweets and chocolate [26].

A total of 124 women (82.7%) reported lifestyle changes following their breast cancer diagnosis, notably adopting healthier eating habits (41.3%), increasing physical activity (27.3%), and reducing alcohol consumption (25.3%). Participants also described changes in their outlook and philosophy of life, including efforts to avoid stress and a preference for ecological and organic food products, as well as hygiene items made from plant-based ingredients.

**Table 1:** Demographic and general characteristics of the study population at the moment of the interview.

Sociodemographic Variables	Study Population (*N* = 150)	
Population area		
Rural area	94 *	62.67%
Urban area	56	37.33%
Education level		
Unschooled	5	3.33%
Primary education	36	24.00%
High school	58	38.67%
University	51	34.00%
**Current** **self-perception of diet compared to other women with BC**		
Quite balanced	78	52.00%
Balanced	55	36.67%
Unbalanced	14	9.33%
Highly unbalanced	3	2.00%
**Current self-perception of diet compared to before diagnosis**		
Balanced	83	55.33%
Quite balanced	46	30.67%
Unbalanced	19	12.67%
Highly unbalanced	2	1.33%
**Sedentary behavior (sitting time ≥ 7 h)**	89	59.33%
**Surgical interventions associated with BC ^1^**	142	94.67%
**Other surgical interventions**	49	32.67%
**Smoking habit**		
Never smoked	82	54.67%
Former smoker	48	32.00%
Current smoker	17	11.33%
Second-hand tobacco smoker ^2^	3	2.00%
**Alcohol consumption**		
Never	76	50.67%
Occasional drinker ^3^	61	40.67%
Current drinker ^4^	7	4.67%
Former drinker ^5^	6	4.00%
**Use of illegal drugs**	5	3.33%
**Dietary supplements (regular consumption)**		
Vitamins and minerals	76	50.67%
None	65	43.33%
Collagen	4	2.67%
Probiotics	3	2.00%
Others	2	1.33%
**Use of sweeteners**		
None	57	38%
Regular sugar	36	24.00%
Stevia or saccharin	32	21.33%
Brown sugar, panela or honey	25	16.67%
**Main type of cooking fat**		
Extra virgin olive oil	143	95.33%
Sunflower oil	5	3.35%
Virgin olive oil	1	0.67%
Butter	1	0.67%

* 37 women in the rural population group came from the metropolitan area municipalities of Granada. ^1^ Any operative procedures for tumor removal (lumpectomy or mastectomy), lymph node assessment, and, when applicable, breast reconstruction. ^2^ A non-smoker who is regularly exposed to second-hand tobacco smoke from others [27]. ^3^ Someone who consumes alcohol sporadically, generally less than once per week or on specific occasions like holidays or social events [28]. ^4^ An individual who drinks at least once per week with regularity [29]. ^5^ An individual who did not consume alcohol in the last 12 months, but who did previously to that [30].

**Table 2:** Lifestyle characteristics of the study population at the moment of the interview.

	Study Population (*N* = 150)		
	Median	P25	P75
**Age** **(y)**	59	51	67
**Weight (kg)**	65	60	75
**Height (cm)**	162	158	165
**Body mass index (kg/m^2^)**	25.08	22.50	29.14
**Residence in Granada (y)**	51	30	60
**Time from diagnosis (y)**	3	2	6
**Last medical check-up (m)**	6	3	12
**Number of Children**	2	1	2
**Food consumption (t/w)**			
• Dairy intake	7	7	14
• Egg intake	3	2	4
• Meat intake	2	2	3
• Meat product intake	3	1	7
• Fish and seafood intake	2	1	3
• Salad and vegetable intake	7	7	7
• Fruit intake	21	14	21
• Nut intake	3	1	7
• Legumes	2	2	3
• Bread intake	7	7	14
• Rice, potato and pasta intake	2	2	3
• Sweets and chocolate intake	0	0	1

cm: Centimeters; m: months; y: years; t/w: times per week; P25, P75: 25th and 75th percentiles.

**Table 3:** Self-reported health-oriented lifestyle changes following BC diagnosis.

	*N*	Percentage
**Adoption** **of any positive change in lifestyle habits**	124	82.67%
**Specific health-oriented changes regarding:**		
• Adoption of healthier dietary habits	62	41.33%
• Increase or improvement in physical activity	41	27.33%
• Reduction or cessation of alcohol intake	38	25.33%
• Improvement in sleeping habits or routines	35	23.33%
• Positive changes in social behavior aimed at enhancing social support	25	16.67%
• Increased attention to food labeling and ingredients	20	13.33%
• Positive changes in life perspective and personal priorities	19	12.67%
• Preference for organic or products perceived as healthier	14	9.33%
• Incorporation of relaxation or mindfulness practices	14	9.33%
• Avoidance of perceived harmful chemicals or toxic exposure	12	8.00%
• Changes in work-related activities to improve health or well-being	6	4.00%
• Reported decline in quality of life despite lifestyle changes	3	2.00%
• Increased engagement in self-care practices	2	1.33%

### 3.2. Qualitative Results

#### 3.2.1. Results from Individual Interviews

The open-ended responses from the individual interviews were analyzed by quantifying the frequency of common responses for each question (main topic) (see Table 4). The participants’ verbatim responses related to these topics, translated into English, are included in the Supplementary Materials S2, Section S1.

**Knowledge of exposure to chemicals and associated health risks** (Table 4—topic 1). Nearly forty percent of the 150 individually interviewed women reported having no specific knowledge about health risks involving environmental factors, including chemical pollutants. Among those with greater awareness, air and water quality were most frequently cited as primary sources of chemical exposure, often linked to agricultural practices, food and livestock production, and industrial activities. Substances of greatest concern included heavy metals, plastics, sulfates, and BPA. While participants recognized the widespread presence of these pollutants, they also expressed a sense of powerlessness and limited ability to control or avoid exposure.

**Information received during the BC disease process** (Table 4—topic 2). Two-thirds of the interviewed women reported not having received any information during their disease trajectory—specifically recommendations aimed at improving their quality of life during and after treatment—from hospitals or primary health centers. Among those who did receive guidance from their physicians, some reported eliminating dairy products, red meat, and sugary foods from their diet. A minority also reported having received information from a nutritionist or a cancer association.

**Interest in receiving a guide including chemical exposure prevention and from whom** (Table 4—topic 3). Most women expressed interest in receiving accurate, high-quality information in the form of a guide, though they showed a clear preference for direct advice from healthcare professionals who could clearly explain risks and preventive measures. They regarded such guidance as more trustworthy than information obtained from the internet or media sources. Notably, some participants indicated a desire to avoid any form of guidance, preferring not to become overly preoccupied with the topic.

**Dietary changes following BC diagnosis** (Table 4—topic 4). Mostly, no significant changes were reported, as participants considered their diets to be already healthy. The most commonly cited adjustments involved greater adherence to the Mediterranean diet, including reduction or elimination of sugar, pastries, meat, sausages, and other processed foods, along with the incorporation of organic and more natural products. Several participants also noted a perceived link between BC and the consumption of specific food groups.

**Changes regarding the manipulation of food and cooking tools** (Table 4—topic 5). Most women (75.30%) reported using either glass or plastic containers to store food. However, many indicated that they had discontinued the use of plastic containers following their BC diagnosis, due to concerns about the potential toxicity of plastic cookware. A small proportion of women indicated that the available information on the topic was overwhelming and difficult to process sufficiently to act.

**Knowledge about food origin** (Table 4—topic 6). Participants frequently reported combining shopping at large supermarket chains (66%) with visits to specialty stores such as greengrocers, butchers, and fishmongers (48.7%). One-third of the participants were not particularly concerned about the origin of their food, although 21.30% expressed interest in locally sourced products, particularly those from Spain, Andalusia, or their own home gardens. Additionally, 9.30% indicated an increased consumption of ecological or organic food products despite them being more expensive.

**Reading different product labels** (Table 4—topics 7 to 9). A large proportion of women (62.70%) reported being inconsistent in reading the labels of household cleaning products, citing difficulties due to small font size and complex language. Additionally, 12% indicated that they routinely used the same products and felt confident in continuing to use them (Table 4—topic 7).

Similar patterns were observed with cosmetic and hygiene products, with 50.70% admitting they did not regularly read labels. The most cited reasons for this included the overwhelming amount of information and difficulties in understanding the content (Table 4—topic 8). Nevertheless, many participants reported a shift toward using higher-quality or more natural products, including those purchased from pharmacies or labeled as organic, natural or vegan. Some had replaced conventional deodorants with healthier alternatives and adopted the habit of reading product labels more frequently. Among these individuals, the primary concerns centered on the presence of parabens, silicones, and sulphates, and their potential role as endocrine disruptors.

Regarding food labeling (Table 4—topic 9), participants’ responses fell into three main categories: those who did not habitually read labels (44%), regular readers (34%), and occasional readers (11.70%). Within the first group, the most frequently cited reason for not reading labels was the difficulty in understanding the information provided.

#### 3.2.2. Results from Focus Group Meetings (FGMs)

Table 5 summarizes the FGM findings condensed into 6 main themes and 26 specific subtopics related to the research interest of this study. Examples of participants’ direct comments on each topic can be found in Supplementary Materials S2, Section S2.

**Theme 1: Risk perception about chemicals and cancer**. Several women reported not perceiving themselves at risk from exposure to hazardous chemicals throughout their lives but expressed increased concern following BC diagnosis. In this respect, some participants linked potential chemical exposure to the use of bleach, cleaning agents, and the presence of parabens in personal hygiene products. Two women identified agricultural and fishing practices as additional sources, mentioning specifically mercury in fish and dichlorodiphenyltrichloroethane (DDT) as a persistent environmental contaminant with year-round exposure risks. Petrol and its strong odor were also commonly associated with a perception of toxicity. Similarly, exposure to tobacco smoke, the presence of plastics in hygiene products and food, and farm fumigation were all identified as potentially hazardous.

**Theme 2: Perceived causes of breast cancer.** Participants in the FGMs attributed BC disease mostly to genetic factors, with a minor contribution from exposure to environmental risk factors, including chemicals. On the other hand, a large majority of participants highlighted the very significant role of emotional stress and psychosocial burden, the lack of personal time, and tobacco use—with alcohol consumption mentioned to a lesser extent.

**Theme 3: Prevention and self-care practices following BC diagnosis.** One of the primary lifestyle changes mentioned by participants following BC diagnosis was an increase in physical activity, engaging in sports such as yoga, Pilates, and rowing. In this respect, several participants highlighted how rowing in teams of BC survivors became not only a physical activity, but a source of emotional healing and empowerment, sharing practical knowledge about living well after cancer. They also highlighted the key role of NGOs in launching and sustaining these initiatives, helping survivors reclaim their well-being through movement, mutual support, and hope.

Participants also reported eliminating refined sugar from their diets, adopting the Mediterranean dietary pattern, and opting for glass containers over plastic for food storage. Additionally, some individuals modified their selection of personal hygiene products, paying closer attention to their ingredients and chemical composition.

**Theme 4: Financial issues and other limitations for adopting protection measures.** Major barriers to adopting healthier lifestyles and protective measures against hazardous chemical exposure include financial constraints, limited health literacy, and restricted access to reliable information on how and where to obtain nutritious foods and personal care products free from harmful substances (e.g., organic items or those without parabens). Additionally, widespread skepticism toward product labeling and marketing claims—particularly those identified items as organic or non-toxic—further hinders informed decision-making.

**Theme 5: Information needs on chemical exposure and BC**. Prior to their BC diagnosis, most participants had not actively sought information related to chemical exposure; only one reported doing so, and exclusively through the internet. Following their diagnosis, individual participants searched for information on specific topics: one focused on plastics, another on food preservatives, and a third on cosmetic and beauty products.

In terms of information sources, participants primarily relied on the internet and television. However, the overwhelming volume of available content often led to feelings of anxiety, particularly due to difficulties in discerning credible and relevant information. Additionally, many participants frequently received and accepted recommendations from family members and friends.

In terms of educational resources, participants expressed a strong interest in receiving guidance on nutrition, cosmetic and hygiene products, and physical activity. They said that this information should be delivered by qualified healthcare professionals, both in person and via a digital platform featuring videos and interactive communication tools. Importantly, they suggested that access to this platform should be introduced at the time of diagnosis to ensure trust in the credibility of the information provided. Finally, participants highlighted the value of including testimonials from women with lived experience of BC, noting that such narratives would offer emotional support and practical insight during the treatment journey.

**Theme 6: Interaction with healthcare providers**. A key concern expressed by several participants, that did not emerge during the individual interviews, refers to the insufficient information provided by healthcare professionals at the time of BC diagnosis. Many reported a lack of understanding regarding the specific characteristics of their cancer type. Participants emphasized that they would have appreciated clearer communication about the severity of the condition, the steps required to manage and overcome the disease, and a comprehensive explanation of the proposed treatment plan.

One participant noted receiving care from an “integrative oncologist,” who offered a more empathetic and holistic explanation of the diagnosis. This included guidance on post-treatment self-care and recommendations for healthy lifestyle habits, although without specific advice on avoiding chemical exposure.

The majority of participants advocated for the establishment of a dedicated health unit designed to address the diverse informational needs of BC patients, including access to psychological support.

## 4. Discussion

To our knowledge, this is one of first studies examining the unmet information needs of BC survivors regarding inadvertent exposure to chemicals present in the environment and everyday products. Although survivors often value personalized and empowering lifestyle-related guidance, existing resources rarely address chemical risks, despite their relevance for disease development, recurrence, and comorbidities.

Our study reveals little awareness among BC survivors of the potential health implications associated with everyday exposure to environmental chemicals prior to diagnosis, alongside an increase in concern following diagnosis. Similarly, Wang et al. (2022), in their systematic review, reported low global levels of awareness and knowledge of BC risk factors (average 40%; 95% CI: 24–56%), with no clear evidence of improvement over time and with chemical exposures not addressed in any instance [31]. In contrast, our study population demonstrated adequate awareness of other well-established BC risk factors, such as smoking and family history. However, high stress levels were frequently highlighted by women in our sample as perceived triggers of BC. Although existing evidence suggests a biologically plausible association, no definitive link has been established, warranting further investigation [32,33].

The group of environmental chemicals mentioned as a threat to human health the most in the present study were those present in air and water and those used in agriculture and food production or packaging. Reference to air quality by participants aligns with evidence showing persistently poor air quality in Granada (Spain) and strong correlations with increased risks of several major diseases, underscoring the city’s substantial environmental burden and the need for targeted public health interventions [34].

The systematic review conducted by Pravednikov et al. (2024) identified 45 studies published between 1985 and 2023 on perceived health risks associated with EDCs, noting that risk interest prior to 2010 focused largely on pesticides associated with agriculture practices, whereas later work increasingly examined compounds such as BPA, phthalates, and parabens [35]. This shift reflects growing evidence of widespread exposure through consumer products, advances in biomonitoring enabling detection of low-dose exposures, and expanding toxicological data on endocrine effects, alongside heightened public and regulatory attention. The review also reported an age-related gradient in risk perception, with older adults showing greater concern than younger individuals, an observation also found in studies focused on the perception of BC risk factors [31,36].

More specifically, participants in our study mentioned heavy metals, plastics, sulphates, BPA and DDT. Similar findings were recorded in a qualitative study of community-dwelling adults from seven European countries within the HBM4EU project, where concerns focused on chemical exposure through food, air pollution, and drinking water [37]. Preservatives and E-numbers were the most frequently cited food-related chemicals, followed by pesticides, herbicides, insecticides, and heavy metals, aligning with our results. Notably, participants of the HBM4EU project did not associate chemical exposure with BC [37], a pattern consistent with the little awareness of chemical-related BC risk observed in our study.

The April 2024 Eurobarometer reported relevant findings on PFAS awareness: only 29% of Europeans—21% in Spain—had heard of PFASs, while 71% had not. Awareness increased to 44% among individuals with higher education levels. After receiving a brief definition of PFASs, more than 80% of respondents expressed concern about the potential health effects of PFAS exposure, indicating that risk perception increased substantially once basic information was provided [38].

Higher educational attainment has been consistently linked to greater BC awareness, as it enhances women’s ability to access, interpret and use health information related to risk factors and prevention. Moreover, higher education is often associated with improved socioeconomic status, which further facilitates access to healthcare resources and preventive services [31,35]. In this regard, the results of a population-based cross-sectional survey among women of reproductive age concluded that women aged 45 to 49, with higher levels of education and a better economic status, as well as those who frequently access information through radio or newspapers, showed a greater degree of awareness about BC and associated risk factors [36].

Many everyday products contain potentially harmful chemicals, including PFASs used in waterproof materials, BPA found in plastics, phthalates present in fragrances, and parabens commonly added to personal care items. These substances, along with volatile organic compounds (VOCs) released from cleaning products and paints, have been associated with cancer, endocrine disruption, and respiratory problems [10,11].

Most participants in the present study, although reporting mostly medium-to-high educational levels, generally paid little attention to the composition of cleaning products, personal hygiene and cosmetic items, arguing difficulties in reading or interpreting labels. Low risk perception in handling cleaning products is a common cognitive bias arising from their routine, everyday use, which fosters a false sense of safety or complacency. Individuals are often unaware of such risks until an accident occurs or a health problem arises and therefore fail to adopt appropriate safety measures [10,39].

Participants in an online survey of everyday products—despite having above-average education, chemical knowledge, and motivation—demonstrated substantial misconceptions about the potential health impacts of harmful chemicals in everyday products [40]. Tariq et al. (2025) reported similar findings in a cross-sectional study of female medical students [41]. While many participants recognized chemical exposure from cosmetics as a potential BC risk factor, most had not examined the specific links between individual ingredients—some of which are EDCs—and the disease [41]. This gap highlights the limits of formal training in conveying practical chemical safety knowledge and underscores the need for clearer risk communication through improved labeling and targeted awareness efforts. It also points to the importance of integrating environmental health topics into medical curricula to better prepare future physicians to provide preventive guidance to women.

Trifunovski et al. (2025) also identified a gap between awareness of EDCs and protective behavior among women aged 18–35 [42]. Although lead was widely recognized as hazardous, this recognition did not translate into avoidance—likely because regulatory bans led participants to perceive lead as a resolved risk. Consistent with the Health Behavior Model, low perceived susceptibility reduced motivation to engage in protective behaviors [42].

Regarding location, evidence suggests that women in urban areas exhibit greater awareness of BC risk factors than those in rural settings, likely due to increased access to awareness programs and health services, higher educational attainment, and greater gender equality [31,36]. Nevertheless, in the context of chemical exposure as a BC risk factor, paradoxical situations may arise in individuals’ receptivity to risk-related information. In our study, although a relatively high proportion of participants expressed a desire to be informed about the risks and preventive measures associated with chemical exposure, some women reported reluctance because such information heightened their anxiety. Self-efficacy—an individual’s belief in their ability to engage in behaviors that reduce environmental risks—strongly shapes these responses. High self-efficacy is associated with perceiving risks as manageable, experiencing less anxiety, and being more willing to engage with preventive guidance. By contrast, low self-efficacy leads individuals to view risks as uncontrollable and to avoid information that could increase feelings of stress or helplessness, particularly when exposure sources appear unavoidable (e.g., high atmospheric pollution levels affecting the city of Granada) or safer alternatives seem inaccessible or unaffordable (e.g., the higher cost of organic foods or natural household products) [43]. Consequently, both the characteristics of environmental pollution and the socioeconomic profile of affected BC survivors become key determinants of whether individuals engage with—or withdraw from—information on chemical exposure prevention.

Consistent with previous evidence [21], more than half of the BC survivors in our study reported prioritizing their health after diagnosis by adopting lifestyle changes such as increasing physical activity—often through group-based activities like rowing—and engaging in practices that support emotional well-being, including yoga. Many participants also described sharing experiences with other survivors as an important source of support during follow-up care. Similarly, the network meta-analysis by Yeganeh et al. (2024) found that psychological interventions combined with increased physical activity produced the greatest improvements in survivors’ quality of life [20]. Broader survivorship research aligns with these findings: Gavili et al. (2024) highlighted supportive care—encompassing social, emotional, and psychological needs—as a critical yet challenging aspect of survivorship, reinforcing the value of comprehensive approaches that address the full range of survivors’ life circumstances [4].

Regarding recognized and reliable sources of information for self-care after diagnosis and for adopting healthier habits in the aforementioned areas, most participants in our study unexpectedly reported receiving no guidance of this kind from healthcare professionals. Their participation in the FGMs further revealed communication about the BC diagnosis, treatment, and follow-up to be poor, unclear, and difficult to understand. In this respect, Niño de Guzmán et al. (2020) and Matheson et al. (2025) similarly report inadequate implementation of clinical guidelines for BC patients among healthcare professionals, underscoring the need to strengthen professionals’ skills, knowledge, and confidence not only regarding the disease itself, but also concerning the evidence related to a wide range of lifestyle behaviors [21,44]. This training, along with the review of BC clinical guidelines, should include clear and accessible information on avoiding exposure to chemicals associated with the disease.

For a communication strategy to be effective and promote meaningful behavioral change, it is essential to adopt a comprehensive approach that integrates multiple methods and behavioral theories, alongside a clear understanding of the target audience’s characteristics and information needs. Evidence in cancer care shows that effective communication must enable patients to understand information, manage emotional responses, and make informed decisions [45]. Furthermore, clinical practice guidelines highlight communication as a key determinant of medical and psychosocial outcomes and emphasize that it can be strengthened through structured [46] public health frameworks also stress the need for evidence-based, audience-centered communication interventions that are tested in real-world settings to ensure their effectiveness and applicability [45]. Similarly, research on oncologists’ communication about modifiable risk factors indicates that clearer, more direct communication is needed to support behavioral change among cancer survivors [47].

In this regard, our findings underscore the need to develop communication campaigns for BC survivors that provide information both in written formats and through websites coordinated by health authorities. These campaigns should combine brief informational brochures with multimedia resources offering comprehensive guidance on managing illness and follow-up care, including aspects related to self-care and diet, that incorporate clear messages for selecting foods and everyday products that minimize exposure to chemicals. The inclusion of videos featuring locally recognized medical professionals or scientists, as well as testimonies from other patients, emerged as a clear priority. Participants also emphasized that such information should be available from the moment of diagnosis and provided continuously throughout the care pathway.

A key issue reported by some BC survivors in our FGMs was the need to improve coordination among existing support services to ensure integrated, continuous care. Although psychosocial, rehabilitative, and exercise-based resources are available, fragmented communication and limited collaboration reduce their accessibility. As noted by Contri et al. (2025), strengthening cooperation among providers and organizing services within a unified, patient-centered pathway can help close gaps, prevent duplication of efforts, and deliver more coherent support throughout survivorship [22].

This study has several limitations. Its cross-sectional design and the intentional, non-probabilistic sampling may limit representativeness, particularly as most participants lived in urban-influenced municipalities within the Granada metropolitan area. Further research is needed to explore the topic in a more diverse sample. Self-reported data may also be affected by recall or social desirability bias, and the smaller number of focus group participants reduces the diversity of qualitative perspectives. Nevertheless, the mixed-methods design, the sizeable sample of breast cancer survivors, and the convergence of findings across interviews and focus groups strengthen the credibility of the results. Importantly, the patterns reported here—regarding lifestyle changes after diagnosis and the categories of information survivors seek—are highly consistent with previous research, supporting the transferability of our conclusions. Moreover, this study provides novel insight as the first to specifically examine breast cancer survivors’ knowledge and informational needs concerning chemical exposure, offering valuable evidence to inform future health communication strategies.

## 5. Conclusions

This mixed-methods study provides novel evidence that BC survivors have limited awareness of everyday chemical exposure and substantial unmet information needs regarding exposure-related risk reduction, despite adequate knowledge of established breast cancer risk factors. Survivors reported perceived gaps in healthcare communication, particularly in relation to environmental health, lifestyle counseling, and product label literacy, which constrained informed decision-making about chemical exposure and long-term self-care. Although diagnosis often prompted positive lifestyle changes, fragmented clinical and community support limited survivors’ ability to translate concern into effective protective behaviors. Integrating clear, actionable environmental health information into survivorship care pathways may strengthen self-efficacy, reduce exposure-related anxiety, and improve overall survivorship outcomes.

## Figures and Tables

**Table 4:** Breast cancer survivors’ most frequent responses regarding open-ended questions from the individual interviews.

Main Topic/Question	Rank	Most Frequent Responses	Percent
Knowledge of chemical pollutants (CP) and their relationship with Breast Cancer (BC)	1	I do not know what CPs are nor their risks on health	39.33%
	2	I know a bit about how CPs can affect health	38.67%
	3	I am aware of the presence of some CPs in the air and water	25.33%
	4	I am aware of some CPs from agriculture, livestock or industrial sources	18.67%
	5	I am aware of the presence of some CPs in personal care products	18.67%
	6	I feel like CPs are everywhere and there’s not much we can do	17.33%
	7	Comment related to pesticides	6.67%
	8	Comments related to heavy metals	6.00%
	9	Comments related to plastics	6.00%
	10	Comments related to sulfates and bisphenol A	5.33%
2. Information received during follow-up care	1	I have not received information during BC follow-up	71.33%
	2	My doctor recommended me to avoid dairy, meat, and sugar	15.33%
	3	I got some general advice from my doctor	8.67%
	4	I got some general advice from a nutritionist	7.33%
	5	I looked for info through media and influencers	7.33%
	6	I have been informed by a cancer association	3.33%
3. Interest in receiving an informative guide including chemical exposure prevention and from whom	1	I am interested due to increased awareness and lack of reliable information	78.67%
	2	I prefer a guide provided by healthcare professionals	29.33%
	3	I am indifferent to receiving a guide or not	9.33%
	4	I don’t really trust info from TV or social media	6.67%
	5	I’d prefer the guide to be given by someone who explains things clearly	4.67%
	6	I prefer that the guide be provided by a BC association	4.00%
	7	I’m not very trusting of authorities	2.67%
	8	I don’t want to receive a guide	2.67%
4. Dietary changes following BC diagnosis	1	I maintain the same eating habits	44.67%
	2	I try to follow the guidelines of the Mediterranean diet better	21.33%
	3	I have decreased sugar consumption	20.67%
	4	I have decreased processed meat consumption	14.00%
	5	I have decreased processed food consumption	13.33%
	6	I have increased organic product consumption	11.33%
	7	I try to choose healthier dishes in restaurants and bars	8.00%
	8	I have decreased pastry product consumption	6.67%
	9	I am aware of the relationship between food and disease	6.00%
5. Changes regarding the manipulation of food and cooking tools	1	I keep using plastic containers	38.00%
	2	I use glass containers	37.30%
	3	I have not changed my way of manipulating food	20.70%
	4	I stopped using plastic containers after diagnosis	14.70%
	5	I use the same cooking tools	6.70%
	6	I found it difficult due to information overload	4.00%
	7	I stopped using Teflon pans	4.00%
	8	I tried to avoid the microwave	2.00%
6. Knowledge about food origin	1	I purchase groceries at the supermarket	66.00%
	2	I purchase groceries in specialized stores	48.70%
	3	I have no concern about food origin	28.70%
	4	I prefer locally or nationally produced food	21.30%
	5	I try to check the origin of the food I consume	18.00%
	7	I try to consume ecological or organic food	9.30%
	8	I have an intermittent interest in food origin	6.00%
	9	I consider organic products expensive	4.70%
7. Habits concerning reading cleaning product labels since BC diagnosis	1	I read cleaning product labels from time to time	62.70%
	2	I use the same cleaning products	12.00%
	3	I have a habit of reading cleaning product labels	9.30%
	4	I do not read cleaning product labels	8.00%
	5	I read labels since COVID-19	3.30%
8. Habits concerning reading cosmetic and hygiene product labels since BC diagnosis	1	I do not read hygiene product labels	50.70%
	2	I consume higher-quality cosmetic and hygiene products	21.30%
	3	I usually read cosmetic and hygiene product labels	14.00%
	4	I read the labels regarding parabens, silicones, sulphates	8.70%
	5	I do not understand the labels	8.00%
	6	I always use the same hygiene products	7.30%
	7	I prefer vegan cosmetic products	2.70%
9. Habits concerning reading food labels	1	I do not read food labels	44.00%
	2	I usually read food labels	34.00%
	3	I have and intermittent habit of reading food labels	11.70%
	4	I read the labels looking for sugar content	8.70%
	5	I read the labels looking for preservative content	8.00%
	6	I read the labels looking for expiration date	7.30%
	7	I use the Yuka mobile App to interpret food labels	6.70%
	8	I read the labels looking for E-numbers	4.00%

**Table 5:** Key findings from focus group discussions: themes and prioritized subtopics.

**Main Themes**	**Subtopics**
**Risk perception about chemical exposure**	▪ Doubt or lack of perception of direct threat ▪ Recognition of specific chemicals (bleach, parabens, plastics, DDT, tobacco, gasoline) ▪ Recognition of past exposure (fumigations, pesticides, air quality pollutants, additives)
2. **Perceived causes of BC**	▪ Emotional stress and psychosocial burden ▪ Personal habits (tobacco, alcohol, diet) ▪ Genetic factors ▪ Environmental and chemical factors (pollutants, endocrine disruptors, diet)
3. **Prevention and self-care practices following BC diagnosis**	▪ Increase physical activity, preferably in the company of other BC survivors ▪ Dietary changes (adopt a Mediterranean diet, reduce sugar, and use glass food containers) ▪ Avoid dyes and use chemical-free cosmetics ▪ Choose foods more carefully and avoid unnecessary additives
4. **Financial issues and other limitations for adopting protection measures**	▪ Financial limitations in accessing healthy products ▪ Difficulty identifying and accessing foods and daily hygiene products free of chemical products ▪ Skepticism towards products labeled as organic and non-toxic
5. **Information needs on chemical exposure and BC**	▪ Interest in seeking information after BC diagnosis ▪ Sources of accurate and well-structured information ▪ Information from life experiences of BC survivors
6. **Interaction with healthcare providers**	▪ Fear and vulnerability at diagnosis time ▪ Demand for clear, comprehensible information about cancer type and prognosis following diagnosis ▪ Demand for professional psychological support ▪ Demand for a specific health unit which provides integrated care for BC patients

## Data Availability

Part of the original contributions from participants in this study, translated into English, are included in the Supplementary Materials S2: Sections S1 and S2. Further inquiries can be directed to the corresponding author.

## Supplementary Materials

The following supporting information can be downloaded at https://www.mdpi.com/article/10.3390/toxics14060456/s1, Supplementary Materials S1: Section S1, Original (Spanish) and translated semi-structured questionnaire adjusted to BC survivor women; Section S2, Focus Group Guide Questions; Supplementary Materials S2: Section S1, Translated discourses of the women from the individual interviews; Section S2, Discourses of the women from the focus groups.

## Abbreviations

The following abbreviations are used in this manuscript:

BC	Breast Cancer
BEUC	The European Consumer Organization
BMI:	Body Mass Index
BPA	Bisphenol A
DDT	Dichlorodiphenyltrichloroethane
EDCs	Endocrine-Disrupting Chemicals
FGM	Focus Group Meeting
PFOA	Perfluorooctanoic Acid
PFASs	Per- and Polyfluoroalkyl Substances
PFOS	Perfluorooctane Sulphonate
POP	Persistent Organic Pollutant
SEOM	Spanish Society of Medical Oncology
