# Supplementary material for: Unmet Information Needs of Spanish Female Breast Cancer Survivors on Chemical Pollutants: A Cross-Sectional Mixed-Method Study
Source: Toxics. 2026 May 23;14(6):456. doi: 10.3390/toxics14060456 (PMC13306574; doi:10.3390/toxics14060456)
Supplement: Supplementary file 1 [file toxics-14-00456-s001.zip › toxics-4214650-supplementary.pdf]

toxics

MDPI

Supplementary Materials

Unmet Information Needs of Spanish Female Breast Cancer Survivors on Chemical Pollutants: A Cross-Sectional Mixed-Method Study

Laura García-Molina, Bibiana Navarro-Matillas, Blanca Riquelme-Gallego, Marina Zenobia Molina-Fernández, José Expósito, Juan Pedro Arrebola and Piedad Martin-Olmedo *

For further information, please contact: piedad.martin.easp@juntadeandalucia.es

Supplementary Materials S1, Section S1.

Original (Spanish) and translated semi-structured questionnaire adapted for breast cancer survivors to obtain information on potential exposure to environmental chemical pollutants before and after a breast cancer (BC) diagnosis. The quantitative questions primarily addressed sociodemographic characteristics and individual lifestyle behaviors, including diet, physical activity, sleep habits, quality of life, and general health status. The open-ended questions explored participants' knowledge of chemicals in general and their potential relationship to human health following exposure.

 https://doi.org/10.3390/toxics14060456

ENCUESTA NUTRICIONAL PARA MUJERES SUPERVIVIENTES DE CÁNCER DE MAMA

DATOS GENERALES

Edad de la mujer:_______años **Altura:**______cm **Peso:**_______________kg **Nacionalidad:**____________________

Tiempo de residencia en Granada:_____**Profesión**: _________

Teléfono:______________ **Correo electrónico**: ____________________________________

Años desde el diagnóstico:_________años **Meses desde la última revisión:**_______meses

Nº hijos nacidos: 0 1 2 3 ó más

Nivel de estudios: Sin estudios Primarios Bachillerato o F.P. Titulación universitaria

¿Cómo considera que es su alimentación actual comparada con la de otras mujeres en su situación?

Equilibrada Bastante equilibrada Poco equilibrada Muy desequilibrada NS/NC

¿Cómo considera que es su alimentación actual comparada con la de antes de su diagnóstico?

Equilibrada Bastante equilibrada Poco equilibrada Muy desequilibrada NS/NC

Actividad física en un día típico (horas totales):

Horas tumbada o dormida: _________

Horas de actividades sentada: _________

Horas de actividades de pie o en movimiento: ____

INFORMACIÓN OBSTÉTRICA

Tipo de embarazo: Único Gemelar Triple o más **Lactancia**: Sí No

Consumo de anticonceptivos: Sí No **Terapia hormonal sustitutiva:** Sí No

Intervención quirúrgica: Sí No **Otras enfermedades relacionadas**: Sí No

Tipo:

INFORMACIÓN SOBRE CAMBIOS DE HÁBITOS DE VIDA

A raíz del diagnóstico, ¿en qué área relacionada con los hábitos de vida ha realizado cambios?:

Alimentación Actividad física Consumo de alcohol y tabaco

Sueño Meditación y relajación

Social Consumo de productos ecológicos

Preocupación o lectura de etiquetado alimentario y de productos de higiene

Información sobre compuestos tóxicos y químicos en diferentes productos

HÁBITOS

**Tabaquismo:**	No	Previo	Activo	Pasivo
**Consumo de alcohol:**	No	Previo	Puntual	Frecuente
**Consumo de drogas:**	No	Previo	Puntual	Frecuente

HÁBITOS ALIMENTARIOS

Frecuencias de consumo: nunca (0) / mensual (1, 2 ó 3) /semana (1, 2, 3, 4, 5 ó 6) /diario (1, 2, 3, 4, 5, 6 ó más)

**Lácteos:**	____________		
**Bebidas vegetales:**	____________	**Frutas:**	____________
**Huevos:**	____________	**Frutos secos:**	____________
**Carnes:**	____________	**Legumbres:**	____________
**Embutidos y fiambres:**	____________	**Pan:**	____________
**Pescados:**	____________	**Arroz, pasta y patatas:**	____________
**Ensaladas y verduras:**	____________	**Bollería y repostería industrial:**	____________

Suplementación de la dieta:

Leche enriquecida en calcio/vitaminas Fibra/Prebióticos Probióticos Yodo/Sal yodada

Ácido fólico/Vitamina B12 Hierro Polivitamínicos y minerales Ninguno

Tipo de grasa más utilizada para aliñar/cocinar:

Oliva virgen Oliva Girasol Mantequilla Margarina

Tipo de edulcorante más utilizado para endulzar alimentos/ bebidas:

Azúcar Miel Fructosa Sacarina Ninguno

https://doi.org/10.3390/toxics14060456

GUÍA DE PREGUNTAS ABIERTAS PARA ENTREVISTAS INDIVIDUALES

1. Conocimiento sobre la relación entre los productos químicos y el cáncer de mama

a) ¿Sabe si la exposición a productos químicos puede afectar a la salud?
b) ¿Cree que puede haber una relación entre los productos químicos y el cáncer de mama?
c) ¿Cree que ha podido estar en contacto con productos químicos que puedan ser perjudiciales? ¿De qué forma? (Por ejemplo, a través de la alimentación, productos de higiene o productos de limpieza.)
d) ¿Ha buscado alguna vez información sobre los posibles riesgos de los productos químicos? ¿Dónde lo ha hecho? (Por ejemplo, redes sociales, libros, artículos, prensa, documentales, organizaciones o personas cercanas.)
e) ¿Conoce a otras personas con cáncer que puedan haber estado expuestas a productos químicos en su entorno?

2. Información recibida durante la enfermedad

a) Durante su enfermedad, ¿le han informado sobre la posible relación entre los productos químicos y el cáncer de mama?
b) ¿Quién le dio esa información?
c) ¿Confía en esa fuente? ¿Por qué?
d) ¿Consultó otras fuentes de información? ¿Cuánto confía en ellas? ¿Por qué?

3. Interés en recibir una guía práctica con recomendaciones

a) ¿Qué tipo de información le habría gustado recibir durante su proceso para sentirse más segura?
b) ¿Y ahora?
c) ¿Cómo le gustaría recibir esa información?
d) ¿Qué le parecería disponer de una guía con recomendaciones prácticas como forma de información?

4. Cambios en la alimentación tras el diagnóstico

a) ¿Ha dejado de comer algún tipo de alimento desde su diagnóstico? ¿Por qué?
b) ¿Dónde suele comprar los alimentos que consume?

5. Cambios en el manejo de alimentos y utensilios

a) ¿Qué tipo de recipientes utiliza para guardar los alimentos?
b) ¿Ha dejado de usar algún utensilio de cocina tras el diagnóstico? ¿Por qué?

6. Origen de los alimentos e información sobre lo que contienen

a) Cuando compra alimentos, ¿le importa de dónde vienen? (Por ejemplo, si son locales o no.)
b) ¿Dónde suele comprar estos alimentos? (Supermercados, tiendas de barrio, directamente a productores, etc.)
c) ¿Suele leer las etiquetas de los productos envasados?
d) ¿Con qué frecuencia consume productos ecológicos?

https://doi.org/10.3390/toxics14060456

7. Cambios en el estilo de vida

a) ¿Ha hecho otros cambios en su estilo de vida desde el diagnóstico?

8. Uso de productos de limpieza

a) ¿Suele leer las instrucciones y las precauciones en las etiquetas de los productos de limpieza?
b) ¿Ha cambiado este hábito después del diagnóstico?

9. Productos de cuidado personal

a) ¿Suele leer las etiquetas de los productos de cosmética o cuidado personal?
b) ¿Ha cambiado este hábito después del diagnóstico?

https://doi.org/10.3390/toxics14060456

SURVEY FOR BREAST CANCER SURVIVORS

GENERAL INFORMATION

Participant age______ Years **Height:______**cm **Weight:________**kg **Nationality:____________Length of residence in Granada:_Occupation:________**

Telephone:**___________Email address**:**____**

Years since diagnosis:_________-years **Months since the most recent follow-up visit:_______**months

Number of children born:__________

Educational level:

No formal education □ Primary education □ Secondary education □

vocational training □ University degree □

How would you rate your current diet compared with that of other women in a similar situation?

Balanced o Fairly balanced □ Slightly unbalanced □ Very unbalanced □

Do not know / No response □

How would you rate your current diet compared with your diet before diagnosis?

Balanced o Fairly balanced □ Slightly unbalanced □ Very unbalanced □

Do not know / No response □

Physical activity on a typical day (total hours):

Hours lying down or asleep:_

Hours spent seated:

Hours spent moving:

OBSTETRIC INFORMATION

Type of pregnancy: Singleton □ Twin □ Triplets or more □

Breastfeeding: Yes □ No □

Use of contraceptives: Yes □ No □

Hormone replacement therapy: Yes □ No □

Surgical intervention: Yes □ No □

Other related conditions: Yes □ No □

Type:

https://doi.org/10.3390/toxics14060456

INFORMATION ON LIFESTYLE CHANGES

Following your diagnosis, in which lifestyle-related areas have you made changes?

Diet o Physical activity □ Alcohol and tobacco use □ Sleep o Meditation and relaxation □

Social life o Use of organic products □

Concern about or reading food and personal care product labels □

Information on toxic and chemical compounds in different products □

HABITS

**Smoking:**	No □	Previous □	Active □	Passive □
**Alcohol consumption:**	No □	Previous □	Occasional □	Frequent □
**Drug use:**	No □	Previous □	Occasional□	Frequent □

DIETARY HABITS

Consumption frequency: never (0) / monthly (1, 2, or 3) / weekly (1, 2, 3, 4, 5, or 6) / daily (1, 2, 3, 4, 5, 6, or more)

**Dairy products:**	**Fruit:**
**Plant-based beverages:**	**Eggs:**
**Meat:**	**Nuts:**
**Legumes:**	**Processed meats and deli meats:**
**Fish:**	**Bread:**
**Rice, pasta and potatoes:**	**Salads and vegetables:**
**Commercial pastries and baked goods:**	

Dietary supplements:

Milk fortified with calcium/vitamins □ Fiber/prebiotics □ Probiotics □ odine/iodized salt □

Folic acid/Vitamin B12 □ Iron □ Multivitamins and minerals □ None □

Type of sweetener most commonly used to sweeten foods/beverages:

Sugar □ Honey □ Fructose □ Saccharin □ None □

https://doi.org/10.3390/toxics14060456

GUIDE FOR OPEN-ENDED QUESTIONS DURING INDIVIDUAL INTERVIEWS

1. Knowledge about the relationship between exposure to chemical pollutants and the development of breast cancer

a. Are you aware of the health effects associated with exposure to chemical pollutants?
b. Do you believe there is a relationship between exposure to chemicals and breast cancer?
c. Do you believe you may have had contact with chemical compounds that could be harmful? Through which routes? (For example, diet, hygiene products, or cleaning products.)
d. Have you ever sought information about the potential hazards of chemical products? Through which sources? Examples include social media, books, scientific articles, the press, documentaries, environmental organizations, or friends.
e. Do you know other individuals who have had cancer and who may have been exposed to environmental chemical products?

2. Information received throughout the course of the disease process regarding the possible relationship between exposure to chemical pollutants and the development of breast cancer

a. Have you received any information during your illness about the possible relationship between these chemical pollutants and breast cancer?
b. Who provided you with this information?
c. How much confidence do you place in this source? Why?
d. Are there any other information sources you consulted during the process? How much confidence do you place in them? Why?

3. Interest in receiving a practical guide with recommendations

a. What type of information would you have liked to receive during the process to help you approach your illness with greater confidence?
b. And at present?
c. How would you like this information to be provided to you?
d. How would you evaluate having a guide containing these recommendations as one of the channels for receiving information?

4. Changes in diet after diagnosis

a. What types of foods have you stopped consuming since your diagnosis? Why?
b. Where do you purchase the foods you consume?

5. Possible changes in food handling and the use of everyday utensils after diagnosis

a. What containers do you use to store food?
b. Is there any kitchen utensil that you decided to stop using after your diagnosis? Why?

https://doi.org/10.3390/toxics14060456

6. Origin of the foods consumed and knowledge of ingredients

a. When purchasing the foods you consume, are you concerned about their origin? For example, whether they are local, regional, or whether origin does not matter.
b. Where do you usually purchase these foods? (For example, supermarkets, specialty stores—such as deli shops, butcher shops, or neighborhood produce stores—or directly from the producer.)
c. Do you read the labels on packaged foods?
d. How often do you consume organic products?

7. Lifestyle changes

a. Have you made any other lifestyle changes since your diagnosis?

8. Use of cleaning products

a. Do you usually read the instructions and precautions on cleaning product labels?
b. Have you changed this habit after the diagnosis?

9. Personal care products

a. Do you usually read the labels of cosmetic or personal care products?
b. Have you changed this habit since the diagnosis?

https://doi.org/10.3390/toxics14060456

Supplementary Materials S1, Section S2.

Focus group guide questions administered by two previously trained members of the research team to collect information on participants' knowledge, practices, and perceptions related to the proposed topic. The interview process began in January 2023, and the final focus group was completed in April 2024.

Focus Group Guide Questions

1. Throughout your life, have you been concerned about daily contact with chemical products? For example, do you read the labels on cosmetic and personal care products?

2. Have you ever perceived any risk associated with contact with chemical products? If so, in what way?

3. Before your diagnosis, had you ever sought information about contact with chemical products? Where did you look for this information? Who provided it?

4. Have you ever wondered whether your illness could be related to these chemical products?

5. How important do you think external factors have been in the development of your illness?

6. What changes did you make in your daily routine after being diagnosed with breast cancer?

7. What barriers have you identified when trying to implement these changes in your life?

8. Finally, imagine that, based on all this information, we could design a guide containing the information that, in your opinion, a woman recently diagnosed with breast cancer would need to know. What format and design should it have? How would you have preferred to receive it?

https://doi.org/10.3390/toxics14060456

Supplementary Materials S2, Section S1.

Translated discourses of the women from the individual interviews.

Knowledge about exposure to chemicals and associated health risks.

"I don't know anything, it's not something I've been worried about it before." (Housewife, 54 years old)

"No, no… you hear a lot of talking, but I have no real knowledge about it." (Physician, 72 years old)

"We are all surrounded by chemicals everywhere, in our food, in the things they added to it, what we inhale, and the cosmetic." (Secondary school teacher, 59 years old)

"Yes, I'm not sure, but many of the products we use could be dangerous to our health: cleaning products, degreasers, and that sort of thing." (Shop assistant, 56 years old)

"Certainly, if vegetables are not properly washed, they may contain pesticides; some fish may contain heavy metals... we must consider also the pollutants associated to the plastic that wraps our food staff. Atmospheric pollution is also important." (University professor, 55 years old)

"[…] I avoid anything with aluminum or aluminum sulfate. And honestly, when I see metallic compounds listed in cosmetic ingredients, it makes me think about the possible health risks. So, I try to steer clear of all that." (University professor, 42 years old)

Information received during the BC disease process.

"No. I have not been informed, but I can deduce that being expose to chemicals might be associated to health problems. That can't be good for the body." (Housewife, 55 years old)

"No, not really. It was at the same time than COVID-19, at the beginning, and almost everything was over the phone (doctor visits), and I felt alone." (Clinic assistant, 60 years old)

"Mostly the information I heard on TV, […] well the doctor told us not to drink milk or eat cheese." (Cashier, 45 years old)

https://doi.org/10.3390/toxics14060456

Interest in receiving a guideline and by whom.

"Yes, why not? As long as it will be provided by a medical center that would give me more confidence, do you know what I mean? I always trust a doctor more than a politician." (Housewife, 53 years old)

"Yes […] Well, preferably if it is provided by professionals from the hospital, because, of course, you find a lot of information on social media, but you don't know if it's true or not. And preferably in a face-to-face mode." (Housewife, 51 years old)

"Yes, I would have appreciated some dietary advice. It would be nice if there would be guidelines […] I would like to receive it in arriving at the hospital when I come for a check-up." (Physician, 55 years old)

"I would get sick going through a guideline. I would worry excessively. In fact, I don't usually read any medicine labels." (Copy shop assistant, 66 years old)

Dietary changes after diagnosis.

"No, because I haven't changed anything important, you know? […] We have not been eaten badly throughout our life, my mum and dad are home cooks and I'm a great cook, I cook every day." (Telephone operator, 46 years old)

"Well, I revised what a Mediterranean Diet means, and I realized that I was eating healthy […] but there were a few things I have changed as they were not too healthy." (Emergency manager, 51 years old)

"I buy organic products; I used to buy them online and now (a supermarket chain) has a section of organic products." (Librarian, 48 years old)

"Regarding diet, I do believe that there is a relationship with the diseases […] I thought: maybe it was my excessive intake of sweets." (Housewife, 54 years old)

"Well, it is true that I eliminate processed food from my meals, because I often look at the labels of the products to try to get away from sunflower oil, added sugars […] And I try to eat fresh products, fresh." (Chemical engineer, 42 years old)

Changes regarding the manipulation of food and cooking tools.

"I store food in both glass and plastic, whatever I can find. Plastic would be worse, wouldn't it?" (Housewife, 74 years old)

"For food preservation glass, and for freezing, plastic." (Nurse, 51 years old)
"Eh, plastic […] it's not made of glass. Yes, it's bad, I knew that." (Housewife, 63 years old)

Knowledge on the food origin.

"I usually buy fish it in the fishmonger of my neighborhood. I buy meat at butcher's sometimes, but occasionally I also go to (a supermarket chain), but never for fish. It inspires me more confidence the neighborhood's fishmonger [...] I try to get bananas from the Canary Islands, [...] I even try to get fish from here (Granada)." (Housewife, 46 years old)

"I buy from local shops, but there are also some products that I buy at the supermarket. The food origin interests me, but it's unavoidable to buy from abroad because of the price of the supermarket basket." (Infant education teacher, 56 years old)

"I don't care about the origin, and I know I should." (Cleaner, 68 years old)

"I try to make sure it's from Spain, or at least, from the European Union." (Housewife, 54 years old)

"Yes, I don't buy it if it's not from Andalusia or Spain [...] and yes, after being diagnosed of breast cancer, I look more into the details [labelling, origin] more." (Scriptwriter, 61 years old)

Additional lifestyle changes after BC diagnosis.

"The philosophy of life and thoughts. That's all [...] I try to do more exercise now." (Commercial textbooks, 54 years old)

"I've gave up smoking." (Housewife, 60 years old)

"I have increased my physical activity because I have more time. I go to the gym, to Zumba, to yoga. I walk every day." (Midwife, 70 years old)

"Try to practice more sport, reduce stress, and go to a psychologist as well." (Administrative assistance, 44 years old)

Reading different products labelling.

"Well, sometimes I do and sometimes I don't." (Housewife, 60 years old)

"Well, sometimes I read them despite the letter are so small." (Cashier, 75 years old)

"Well, because of the pandemic (COVID-19), I make sure that they are disinfectants, I didn't do that before." (Teacher, 47 years old)

"Not really, because the more I know, the worse it gets." (Shop owner, 52 years old)

https://doi.org/10.3390/toxics14060456

"Yes, I have changed that. Higher quality products, from the pharmacy, neutral pH for the skin. The doctor said so, too. I usually get very itchy on my back, because of the wound on my breast." (Housewife, 78 years old)

"At first, I used to check cosmetic labels because I was really concerned about it. But then I realized it's almost impossible to avoid these substances. I've bought products thinking they were natural or additive-free—maybe they don't have parabens, but when you look closer, they contain other potentially carcinogenic ingredients. Honestly, it feels like a losing battle these days, especially when it comes to endocrine disruptors." (Cardiologist, 53 years old)

"Yes, yes, I do. I try to avoid, the less chemical the best, using vegan and those from natural sources." (Housewife, 54 years old)

"No, because I don't understand anything." (Housewife, 79 years old)

https://doi.org/10.3390/toxics14060456

Supplementary Materials S2, Section S2.

Discourses of the women from the focus groups.

Theme 1: Risk perception about chemicals and cancer

"I'm allergic to bleach, so I started looking for products that don't have it. I also check shampoo labels to make sure they're free of parabens." (Pt 5-G1)

"After this (BC diagnosis) I have decided not to color my hair." (Pt 6-G1)

"I didn't feel it as a direct threat, but as something that affect to others." (Pt 4-G2)

"I always thought that it could be my turn (of suffering cancer) any moment. Until 1960, DDT was used on a massive scale. And I was aware that we were very exposed to it." (Pt 3-G2)

"Not an imminent threat of danger." (Pt 1-G1)

"[…] What do we do with toothpaste that contains plastic?" (Pt 5-G1)

"I have noticed a strong cough when getting in contact with some chemicals, especially tobacco." (Pt 2-G2)

"My parents were from rural areas, and when fumigating, it smelled a lot, so we used to go into the house, which it is a symbol that it is dangerous." (Pt 5-G2)

"Petrol, always. When I used to travel by bus or boat, for example, I got sick from the petrol smell." (Pt 1-G2)

Theme 2: Perceived causes of breast cancer

"There is a genetic predisposition, and there are external factors that help it to manifest itself, but genetic predisposition alone is not enough." (Pt 2-G1)

"(Chemical contaminants) It's in the environment, it's not the main cause, but something contributes." (Pt 1-G1)

"I think it's more about genetics." (Pt 6-G1)

"I think the emotional stress issue is the most relevant factor, I think that's what changed my body." (Pt 4-G1)

"I give more weight to mental stress than to environmental exposure factors." (Pt 5-G1)

"I think the most determinants factor was smoking. I was a smoker for 20 years, and even though I quit smoking 30 years before I was diagnosed, I think it had a lot to do with it." (Pt 2-G1)
"I associate it with alcohol." (Pt 1-G1)

"I have associated it with stress. I spent years when I had no time for anything. Years working alone, taking care of parents, children. […]" (Pt 5-G2)

Theme 3: Prevention and self-care practices following BC diagnosis

"I started doing yoga and Pilates." (Pt 6-G1)

"I started rowing after that, and it's really helped my arm. But it's also been great for sharing stories, tips, and experiences with others BC survivors. The support from breast cancer association in facilitating all that has meant a lot to me". (Pt 3-G1)

"I gave up sugar and refined salt, and try to eat healthy." (Pt 5-G1)

"Since diagnosis, I look at paraben-free lotions or natural soap." (Pt 5-G1)

"I follow the Mediterranean Diet, and use more natural hygiene products." (Pt 4-G1)

"I've included more vegetables and physical activity. Regarding canned food, I try to buy it in glass." (Pt 6-G2)

"I look at additives. I don't know what to eat, chicken is full of hormones." (Pt 5-G2)

"I started to look at labels of lotions and nail polishes after diagnosis, using only natural ingredients, although I am not sure about it". (Pt 1-G2)

Theme 4: Financial issues and other limitations for adopting protection measures

"It was really hard to find stuff without parabens. I've finally come across an organic shop, but before that, I could spend hours wandering around [supermarket name] *trying to find those kinds of products. I love fish, but it's pricey, and my budget doesn't really let me buy organic food or eat as much fish as I'd like."* (Pt 5-G1)

"Not knowing, ignorance, lack of knowledge about ecology." (Pt 1-G1)

"There are many people for whom eating healthy is not affordable. Give me money and don't give me advice. [...] The economy conditions people's health." (Pt 4-G2)

"For example, it is said that extra virgin olive oil helps prevent relapses, but right now I just can't get my hands on it—it's far too expensive." (Pt 1-G2)

Theme 5: Information needs on chemical exposure and BC

"I did not search before diagnosis." (Pt 1-G1)

"I've tried searching online, but honestly, it's overwhelming. There's so much information out there that I don't know what I can trust". (Pt 6-G2)

"After listening to [a well-known professor from Univ. Granada expert in endocrine disruptors], *saying that plastics are endocrine disruptors, I started to look for information."* (Pt 4-G1)

"Some news in TV about the products (chemical products)." (Pt 4-G2)

Theme 6: Demand for quality, accessible and structured information

"A reference platform, indicated by the health professionals, available and accessible on the Internet and supported by professionals. [...]. I wish there was a testimonial from a woman who has already passed BC." (Pt 1-G1)

"Any guidance would be important, including information about the disease, because at the diagnosis, they tell you words you don't understand [...], a guideline containing information referring to recommended dietary guidelines, physical activity you can do (what and where), etc." (Pt 5-G1)

"A platform is good, but a physical support person is also very necessary. I would have appreciated that in addition to physical care, I would have liked psychological support and advice on exercise

guidelines. A YouTube talk that explains basic things about hygiene, cleanliness, diet. […] To include in the platform testimonials from women who have overcome the disease." (Pt 3-G1)

"I would have liked to know which foods could be not adequate for my disease and which ones I can consume […]" (Pt 1-G2)

"It would be very good to have a small information booklet given to you at the time of diagnosis, and after treatment, a series of accompanying sessions. To set up a specific department to help breast cancer patients, with multidisciplinary support." (Pt 6-G2)

"Receive information at the time of diagnosis. Receive reliable websites that allow us to check with confidence. The Internet is full of information, but you don't know where to go. To be told more about nutrition. There is no nutritional guidance, and I think it is important that this is incorporated. A sort of question-and-answer site run by different experts." (Pt 4-G2)

Theme 7: Interaction with Healthcare providers

"More empathy from healthcare professionals. I had three nurses and every time I had a problem, they looked for solutions adjusted to your particular situation. Empathy on the message, is very important. […] The oncologist did not even want to see me." (Pt 1 –G1)
"Why can't we get an endocrinology/nutrition appointment to check us once a year?" (Pt 5 –G1)
"I would have liked to receive guidance on natural remedies that are contraindicated for my condition, […] The oncologist does not know how to respond to these questions." (Pt 1 –G1)

"I missed empathy, as well as information about the treatment I was undergoing and its consequences." (Pt 3 –G1)

"In retrospect, I believe I would have needed psychological support. […] Establishing a specific department to support breast cancer patients, with a multidisciplinary approach. The oncologist is not available to provide this type of counseling; a more specialized space is necessary." (Pt 6 –G1)

"It would be beneficial to have access to a psychologist or therapists at the time of diagnosis, to help patients with the impact of the diagnosis. […] Alongside the oncologist or gynecologist, there should be a counseling unit that provides guidance, helps patients understand what is happening to them, and offers emotional support." (Pt 5 –G2)

https://doi.org/10.3390/toxics14060456
